# Manual annotation of *Drosophila* genes: a Genomics Education Partnership protocol

**DOI:** 10.12688/f1000research.126839.1

**Published:** 2022-12-23

**Authors:** Chinmay P. Rele, Katie M. Sandlin, Wilson Leung, Laura K. Reed

**Affiliations:** 1 Department of Biological Sciences, The University of Alabama, Tuscaloosa, Alabama, 35487, USA; 2 Department of Biology, Washington University in St. Louis, St. Louis, Missouri, 63130, USA

**Keywords:** comparative genomics, Course-based Undergraduate Research Experience, CURE, structural gene annotation

## Abstract

Annotating the genomes of multiple species allows us to analyze the evolution of their genes. While many eukaryotic genome assemblies already include computational gene predictions, these predictions can benefit from review and refinement through manual gene annotation. The Genomics Education Partnership (GEP; https://thegep.org/) developed a structural annotation protocol for protein-coding genes that enables undergraduate student and faculty researchers to create high-quality gene annotations that can be utilized in subsequent scientific investigations. For example, this protocol has been utilized by the GEP faculty to engage undergraduate students in the comparative annotation of genes involved in the insulin signaling pathway in 27 *Drosophila* species, using *D. melanogaster* as the reference genome. Students construct gene models using multiple lines of computational and empirical evidence including expression data (e.g., RNA-Seq), sequence similarity (e.g., BLAST and multiple sequence alignment), and computational gene predictions. Quality control measures require each gene be annotated by at least two students working independently, followed by reconciliation of the submitted gene models by a more experienced student. This article provides an overview of the annotation protocol and describes how discrepancies in student submitted gene models are resolved to produce a final, high-quality gene set suitable for subsequent analyses. The protocol can be adapted to other scientific questions (e.g., expansion of the *Drosophila* Muller F element) and species (e.g., parasitoid wasps) to provide additional opportunities for undergraduate students to participate in genomics research. These student annotation efforts can substantially improve the quality of gene annotations in publicly available genomic databases.

## Introduction

**Figure 1.**
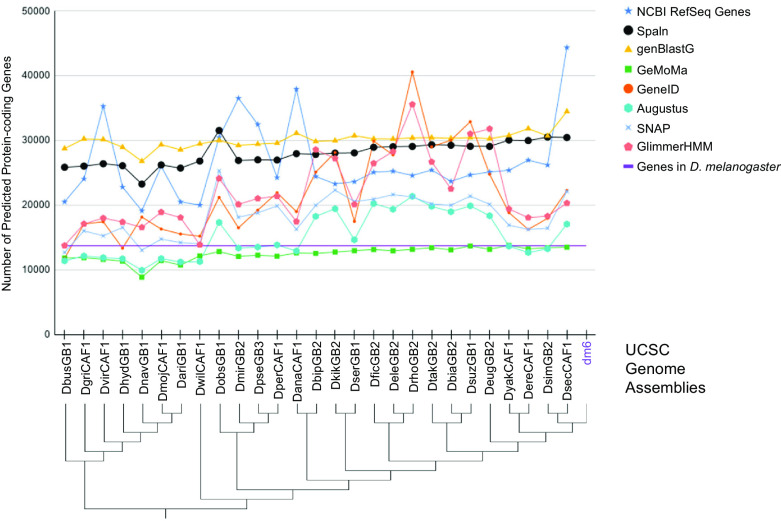
Number of protein-coding genes predicted by different gene predictors for the 27 *Drosophila* species analyzed for the Pathways Project. The number of predicted genes can show large variations across algorithms (algorithm information in extended data ^4^) and species, particularly for gene predictors using sequence similarity to genes in a reference species as their primary source of evidence. Some algorithms consistently either predict more (e.g., genBlastG) or less (e.g., GeMoMa) genes than the number of *D. melanogaster* genes as curated by FlyBase (purple line). Prediction differences can partly be attributed to some algorithms predicting a single isoform in a genomic region (e.g., GeneID), while others predict multiple isoforms per genomic region (e.g., genBlastG, Spaln). The genome assemblies indicated in the cladogram ^5^^,^^6^ correspond to those listed in the “UCSC Assembly” column of Table 1.

Genome annotation requires assessing and integrating multiple lines of computational and empirical evidence. Several computational pipelines have been developed (e.g., BRAKER and MAKER) for constructing an initial set of structural gene annotations for eukaryotic genomes. ^1^^,^^2^ Accuracy of the gene models produced by gene prediction algorithms depends on multiple biological (e.g., genome size, ploidy, repeat density, and complexity of the transcriptome) and technical factors (e.g., quality of the genome assembly, evolutionary distance from the reference species, and availability of transcriptome data). ^3^ These factors can contribute to differing numbers of gene predictions in closely related species ( Figure 1).

Evidence-based gene prediction algorithms (e.g., Gnomon ^7^) use extrinsic evidence, such as RNA sequencing (RNA-Seq) data derived from the target species, and sequence similarity to proteins from a reference species, to predict genes within the target assembly. The advent of high-throughput RNA sequencing technologies have led to substantial improvement in the quality of gene annotations, ^8^^,^^9^ particularly for species that lack high-quality gene annotations from a closely related reference species; however, the efficacy of assembling transcripts from RNA-Seq data depends on transcript expression levels in the specific developmental stages and tissue types that are sampled. ^10^ Long-read RNA sequencing technologies (e.g., Iso-Seq by Pacific Biosciences and Direct RNA sequencing by Oxford Nanopore) can produce reads that span the entire transcript, which facilitates identification of alternative splicing patterns and characterization of different gene isoforms. There are, however, several challenges for producing high quality long-read data including the robustness of RNA extraction methods, bias towards short transcripts, low sequencing throughput, and low read accuracy (reviewed in Ref. 11). Additionally, past studies have shown that transcriptomes constructed from long-read RNA-Seq data have high sensitivity but low precision. ^12^

Consequently, despite recent advances in gene prediction algorithms and the increasing availability of RNA-Seq data, gene predictions produced by computational algorithms can still benefit from manual review and refinement. ^13^^,^^14^ This article describes a protocol, developed by the Genomics Education Partnership (GEP; https://thegep.org), to engage undergraduate students in the comparative annotation of protein-coding genes involved in the insulin signaling pathway (ISP) across 27 species of *Drosophila*, using *D. melanogaster* as the reference species ( Table 1).

**Table 1.** Genome assemblies and RNA-Seq data for the comparative analysis of ISP genes in 27 *Drosophila* species and the reference species *D. melanogaster.* For each UCSC Assembly, the table shows the corresponding NCBI RefSeq Accession Numbers, species names, and BioProject Accession Numbers for the RNA-Seq data.

UCSC Assembly	NCBI GenBank Assembly Accession	Species Name	NCBI BioProject Accession Numbers for RNA-Seq data
DbusGB1	GCA_001277935.1	*D. busckii*	PRJNA274996
DgriCAF1	GCA_000005155.1	*D. grimshawi*	PRJNA317989
DvirCAF1	GCA_000005245.1	*D. virilis*	PRJNA200701
DhydGB1	GCA_002780465.1	*D. hydei*	PRJNA373926
DnavGB1	GCA_001654015.1	*D. navajoa*	No RNA-Seq data available
DmojCAF1	GCA_000005175.1	*D. mojavensis*	PRJNA200701
DariGB1	GCA_001654025.1	*D. arizonae*	PRJNA395148
DwilCAF1	GCA_000005925.1	*D. willistoni*	PRJNA388952
DobsGB1	GCA_002217835.1	*D. obscura*	PRJDB4576
DmirGB2	GCA_000269505.2	*D. miranda*	PRJNA77213
DpseGB3	GCA_000001765.2	*D. pseudoobscura*	PRJNA200701
DperCAF1	GCA_000005195.1	*D. persimilis*	PRJNA388952
DanaCAF1	GCA_000005115.1	*D. ananassae*	PRJNA200701, PRJNA72165, PRJNA257286, PRJNA388952
DbipGB2	GCA_000236285.2	*D. bipectinata*	PRJNA63469
DkikGB2	GCA_000224215.2	*D. kikkawai*	PRJNA63469
DserGB1	GCA_002093755.1	*D. serrata*	PRJNA355616
DficGB2	GCA_000220665.2	*D. ficusphila*	PRJNA63469
DeleGB2	GCA_000224195.2	*D. elegans*	PRJNA63469
DrhoGB2	GCA_000236305.2	*D. rhopaloa*	PRJNA63469
DtakGB2	GCA_000224235.2	*D. takahashii*	PRJNA63469
DbiaGB2	GCA_000233415.2	*D. biarmipes*	PRJNA63469
DsuzGB1	GCA_000472105.1	*D. suzukii*	PRJNA221549
DeugGB2	GCA_000236325.2	*D. eugracilis*	PRJNA63469
DyakCAF1	GCA_000005975.1	*D. yakuba*	PRJNA200701
DereCAF1	GCA_000005135.1	*D. erecta*	PRJNA414017, PRJNA264407
DsimGB2	GCA_000754195.3	*D. simulans*	PRJNA200701
DsecCAF1	GCA_000005215.1	*D. sechellia*	PRJNA205470, PRJNA414017
dm6	GCA_000001215.4	*D. melanogaster*	PRJNA481740

**Figure 2.**
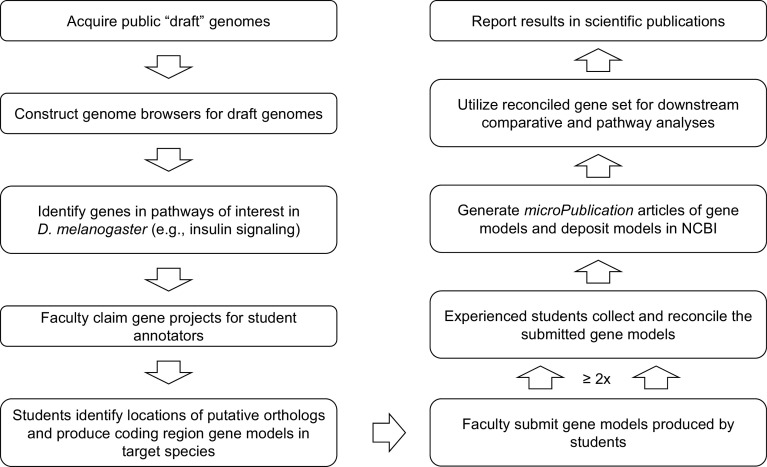
Gene Model Creation Workflow. Summarized workflow for annotation and reconciliation to produce a set of high-quality gene models suitable for comparative and pathway analyses.

As of August 2022, GEP students from 79 institutions have used this annotation protocol to construct 2,101 gene models across 27 *Drosophila* species. Despite differing in instructional settings and teaching modalities, this protocol ensures that GEP students use a uniform standard to construct gene models that are best supported by the available evidence. As an additional level of quality control, each gene is annotated by at least two students working independently, and the submitted gene models are then reconciled by an experienced student using the Apollo genome annotation editor. ^15^ Reconciled gene models will typically be described in *microPublication* articles ^16^ and submitted to the NCBI Third Party Annotation (TPA) database. ^17^ Researchers can utilize the high-quality, manually curated gene models constructed by GEP students to investigate the evolution of genes and genomes ( Figure 2).

## Methods

### Overview of the coding region annotation protocol

*Drosophila* researchers and curators at FlyBase have produced high-quality, comprehensive gene annotations for *D. melanogaster* based on a large amount of genetic and sequencing data. ^18^ Our protocol utilizes the gene annotations from *D. melanogaster* (reference species) to facilitate annotation of the protein-coding sequence (CDS) of orthologous genes in other *Drosophila* species (target species). In the absence of compelling evidence (e.g., RNA-Seq data) indicating significant differences in the gene model, the proposed gene model in the target species minimizes the number of changes compared to the ortholog in the reference species (i.e., construct the most parsimonious gene model assuming evolutionary conservation).

In order to generate manual annotations for the CDS of a gene in the target species, we need to (1) identify the ortholog of that gene from *D. melanogaster* in the target species using sequence similarity and local synteny, (2) determine the structure and approximate coordinates of each isoform and their coding exons, and then (3) refine those coordinates for each isoform. The key analysis steps are also summarized in the Results section and in the Annotation Workflow for the Pathways Project. ^19^ A walkthrough illustrating each step of the annotation protocol from the perspective of a naive student annotator on an example gene is both available on the Pathways Project page of the GEP website ( https://thegep.org/pathways/) and in Ref. 20. Here we highlight the essential conceptual steps the student annotator will follow.

The annotation and reconciliation protocols described below utilize multiple bioinformatics tools that are briefly summarized in the “Data (and Software) Availability” section.

**CDS annotation procedure**

***Project claiming***

GEP faculty members select at least one *D. melanogaster* gene involved in the ISP (i.e., target gene in the reference genome) and one or more of the 27 *Drosophila* species (i.e., target species) for their students to annotate. Each gene project includes an estimated difficulty level based on the number of isoforms, number of coding exons, and evolutionary distance from the reference genome. Faculty members can take the estimated difficulty of the gene projects into consideration when selecting projects that best suit the pedagogical goals of their courses, the amount of class time devoted to the annotation project, the academic levels of their students, and specific interests in the biological function of a gene. For example, faculty members might select the same gene in multiple *Drosophila* species for their students to annotate (working individually or in groups) in order to teach students about conservation relative to divergence time.

***Identify the ortholog***

Ortholog assignment in the target species is based on the analysis of protein sequence similarity and local synteny (i.e., relative gene order and orientation within a syntenic chromosomal region) compared to the reference species. ^21^^,^^22^ The key analysis steps for identifying the ortholog are also summarized in the extended data. ^23^

To identify the ortholog of the target gene, the student annotator examines the genomic neighborhood surrounding the gene in both the reference species and the target species using the GEP UCSC Genome Browser (further described in the Software section below; https://gander.wustl.edu). This analysis includes identifying the nearest two upstream and downstream genes and their orientations relative to the target gene. The local synteny analysis also includes any nested genes in the locus surrounding the putative ortholog in the target species.

Locating the putative ortholog requires the student to obtain the protein sequence for the target gene in the reference species from the Gene Record Finder, and use it as the query to perform a *tblastn* search against the genome assembly of the target species via NCBI Web BLAST ( https://blast.ncbi.nlm.nih.gov/Blast.cgi). This protocol uses the *tblastn* program to compare the protein sequence query against a nucleotide database because amino acid sequences show higher sequence conservation than nucleotide sequences across evolutionary time. In addition, due to the degeneracy of the genetic code, sequence similarity searches at the amino acid level are more sensitive than searches at the nucleotide level. ^24^^,^^25^ There are three possible outcomes of their *tblastn* search: (1) zero matches, (2) one match, or (3) more than one match. If there is a single high-quality match, that match is a good candidate for being the ortholog.

The *tblastn* search may report zero significant matches even if the ortholog exists in the target species due to gaps or misassemblies in the genome assembly or the lack of sequence conservation between orthologs. In the latter case, the two genes upstream and downstream of the target gene in the reference species will be used to infer the location of the target gene in the target species (i.e., local synteny). The student first identifies the orthologs to the genes flanking the target gene in the target species. If there is a locally syntenic region in the target species, then there will likely be an additional feature located between the flanking orthologs in the target species. This additional feature can then be considered as the likely ortholog to the target gene in the target species based on local synteny. Orthology should be confirmed using a *blastp* search of the predicted protein sequence of the feature in the target species against a database of annotated proteins for the reference species. If the location and candidate for the ortholog cannot be established using this strategy, then the ortholog may be absent from the genome assembly for the target species.

If the *tblastn* search reports two or more significant matches, then the ortholog assignment will be based on parsimony with the reference species. The match with the lowest E-value, highest percent identity, and highest alignment coverage to the target gene will be assigned as the putative ortholog in the target species. The student then confirms the putative ortholog assignment derived from the best *tblastn* match via local synteny by comparing the genes surrounding the target gene in the target and reference species. If there is no match for neighboring genes in an evolutionarily diverged species but a single match exists for the target gene, then the ortholog assignment will be determined by BLAST searches using the Reciprocal Best Hits (RBH) strategy. ^26^

**Figure 3.**
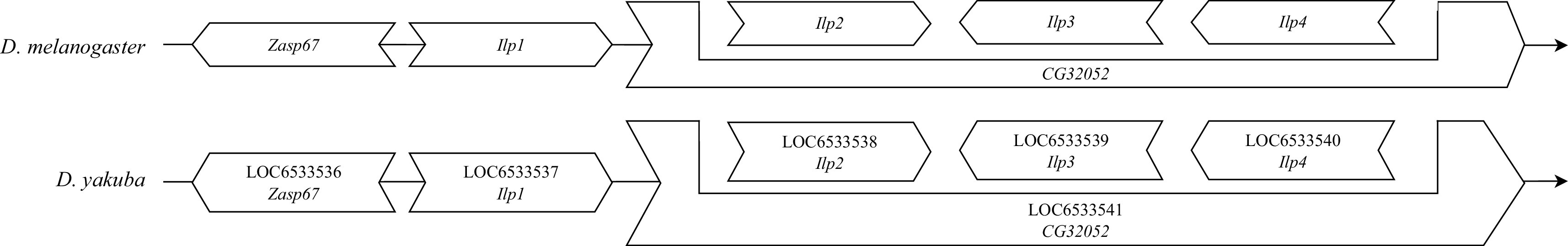
Local synteny analysis of the *Ilp2* gene in *D. yakuba.* Schematic of genomic neighborhood surrounding the *Ilp2* gene in *D. melanogaster* and *D. yakuba*, showing that *Ilp2*, *Ilp3*, and *Ilp4* are nested within *CG32052* in both species.

Figure 3 shows an example of how the student can use local synteny to establish the ortholog of the target gene *Ilp2.* The coding region of the same two genes are located upstream (i.e., *Zasp67* and *Ilp1*) and downstream (i.e., *Ilp3* and *Ilp4*) of *Ilp2* in *D. melanogaster* and *D. yakuba.* In addition, *Ilp2, Ilp3*, and *Ilp4* are all nested within the coding span of *CG32052* in both species, which further supports the hypothesis that the genomic neighborhood is conserved, and thus, the ortholog of *Ilp2* has likely been identified.

***Identify the approximate coordinates of each coding exon***

Once the student identifies the putative ortholog, they begin constructing the gene model by separately mapping each coding exon to determine their approximate locations and their reading frames in the target genome using the “Align two or more sequences” ( *bl2seq*) feature provided by NCBI Web BLAST.

For each isoform of the target gene in *D. melanogaster* (reference genome), they perform *tblastn* searches of each coding exon of the isoform (query) in *D. melanogaster* against the scaffold which contains the putative ortholog of the target gene in the target species (subject). The amino acid sequence for each coding exon in the *D. melanogaster* isoform is obtained from the Gene Record Finder (further described in the Software section below). The scaffold in the target species, which contains the putative ortholog of the target gene, is identified by the “Identify the ortholog” step above. For scaffolds larger than 10 Megabases (Mb), the “From” and “To” fields under “Subject subrange” in the NCBI *tblastn* search interface are used to limit the size of the search region to the approximate location of the target gene (inferred from results of the “Identify the ortholog” step).

The default *tblastn* search parameters for NCBI Web BLAST are used in this search except for the following parameters:

1. Select the “Align two or more sequences” checkbox
2. Specify a subject subrange that corresponds to the approximate location of the target gene estimated at the “Identify the ortholog” step
3. Select “No adjustment” under “Compositional adjustments”
4. Uncheck the “Low complexity regions” filter (under “Filters and Masking”)

At the end of this process, the student usually identifies a collinear set of coordinates for most of the coding exons of the isoform. Results of the *tblastn* searches provide further supporting evidence for the ortholog assignment and provide anchors from which to define the search regions for small or weakly conserved coding exons.

***Refine coding exon coordinates***

Since *tblastn* only aligns to complete codons, the BLAST alignments will not include partial codons adjacent to the splice junctions. In addition, *tblastn* does not take the locations of potential splice sites into account when it generates the alignment. Consequently, other lines of evidence (e.g., computational gene predictions, RNA-Seq data) must be used to refine the start and end coordinates of each coding exon. The student manually refines the collinear coding exon coordinates identified above through visual inspection of the region in the Genome Browser utilizing the RNA-Seq track, other homology-based alignment algorithms, and gene predictions, as well as comparing the sequence to other *Drosophila* species based on whole genome multiple sequence alignments (more details about the tracks used can be found in the extended data). ^4^

If available, the RNA-Seq data can provide empirical support for the proposed gene model in the target species. RNA-Seq data for the target species were obtained from the NCBI Sequence Read Archive ( Table 1 ^27^). The RNA-Seq reads were mapped against the target genome using HISAT2, ^28^ and putative introns were inferred from the alignments of spliced RNA-Seq reads using the *junctions extract* command provided by RegTools. ^29^

**Figure 4.**
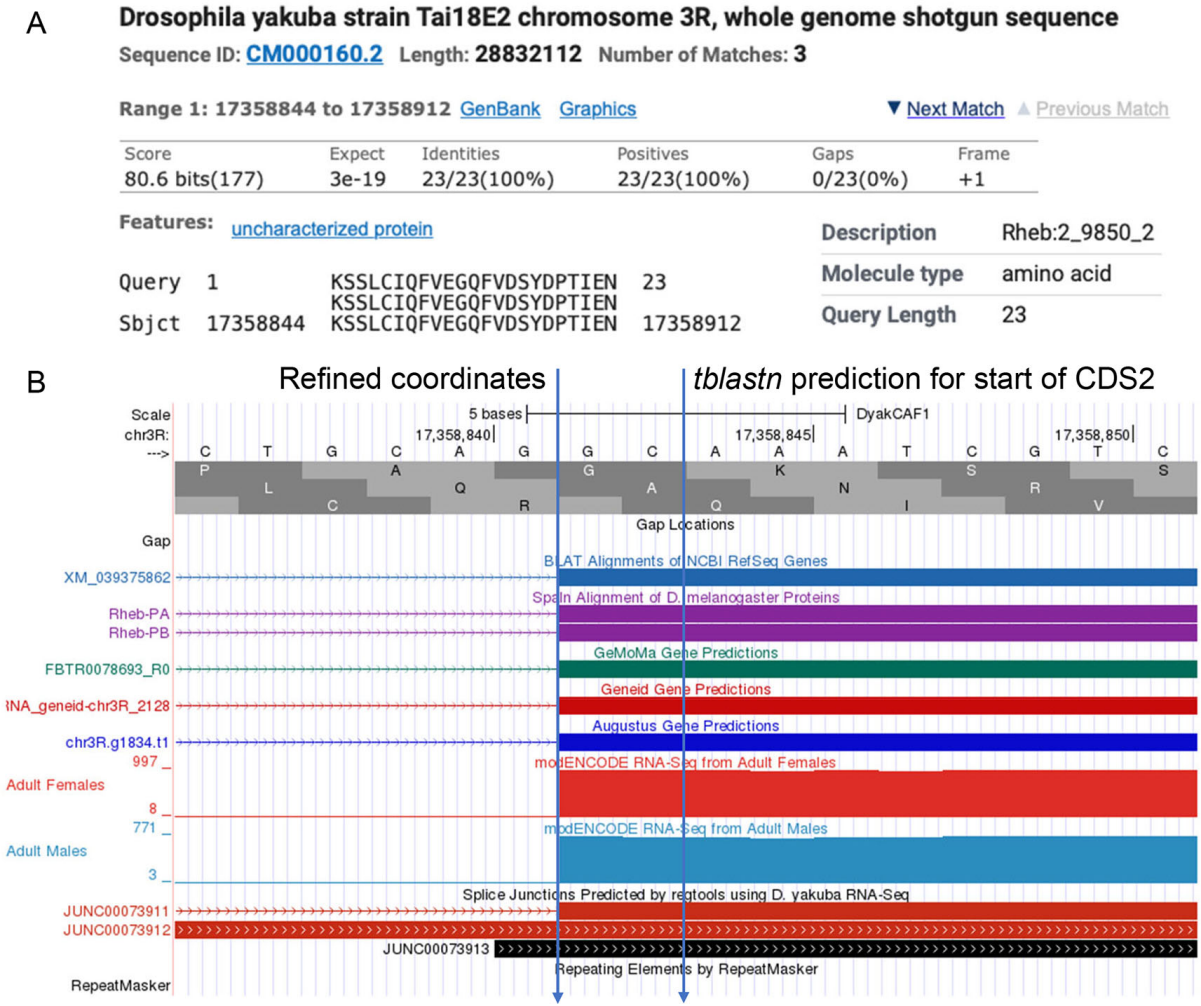
Refined Coordinates for CDS 2 of *Rheb* in *D. yakuba.* (A) The *tblastn* alignment for the *D. melanogaster* CDS 2_9850_2 (query) against the *D. yakuba* scaffold CM000160.2 (chr3R) placed the start of the CDS at 17,358,844 in frame +1. (B) Examination of the other lines of evidence (e.g., RNA-Seq read coverage, splice junction predictions, gene predictions, protein alignments) using the Genome Browser placed the start of the coding exon at 17,358,842. Since there are two nucleotides (GC; blue lines) prior to the first complete codon (AAA) that codes for first amino acid in the *tblastn* alignment (K), CDS 2_9850_2 in *D. yakuba* has a phase 2 splice acceptor site relative to reading frame +1.

When defining the intron coordinates, canonical splice sites (i.e., GT donor and AG acceptor) are adhered to unless there is good evidence to the contrary, such as spliced RNA-Seq reads from the target species and conservation of non-canonical splice sites across the clade. The student also needs to ensure the donor and acceptor sites have compatible phases (i.e., the sum of the phases of the donor and acceptor sites of an intron is either zero or three) in order to maintain the open reading frame after splicing. A more detailed version of the workflow for refining the coding exon coordinates, from the perspective of a naive student, can be found within extended data. ^30^ Figure 4 gives an example coordinate refinement to account for the complete exon boundaries considering RNA-Seq data and splice site compatibility, and Table 2 shows the comparison of the approximate coding exon coordinates determined by the *tblastn* searches with their refined counterparts for the *Rheb* gene in *D. yakuba.*

**Table 2.** Refining approximate *tblastn* coordinates for coding exons of *Rheb* in *D. yakuba* (DyakCAF1). Comparison between the *tblastn* and refined coordinates for the model of *Rheb* in *D. yakuba.* Note that in most cases, the coordinates identified by the *tblastn* search are adjusted by a few nucleotides (difference columns) via visual curation to account for incomplete codons and over-extensions of the *tblastn* alignment.

Exon Number	Approximate coordinates from *tblastn* search		Refined exon coordinates		Difference (refined - *tblastn*)	
	Start	Stop	Start	Stop	Start	Stop
1	17,358,666	17,358,713	17,358,666	17,358,714	0	1
2	17,358,844	17,358,912	17,358,842	17,358,913	-2	1
3	17,359,013	17,359,216	17,359,011	17,359,218	-2	2
4	17,359,279	17,359,407	17,359,278	17,359,407	-1	0
5	17,359,470	17,359,559	17,359,470	17,359,559	0	0

***Verify gene model***

The student uses the Gene Model Checker to verify that the refined coordinates for the gene model in the target species satisfy the biological constraints for protein-coding genes in most eukaryotes and reflect the gene structure of the *D. melanogaster* ortholog. The dot plot and protein alignment identify differences between the proposed gene model and the *D. melanogaster* ortholog, and they help to verify that their proposed gene model is the most parsimonious compared to the *D. melanogaster* ortholog. For more information on the Gene Model Checker, refer to the “Data (and Software) Availability” section.

The student repeats the “Identify the approximate coordinates of each coding exon” and “Refine coding exon coordinates” steps to construct gene models for each unique protein-coding isoform of their target gene and then verifies them with the Gene Model Checker.

***Final submission***

The student submits a file containing the coordinates of the coding exons for all isoforms in the Generic Feature Format (GFF), a file containing the transcript sequence for the coding region of all isoforms in FASTA format (FNA), and a file containing the peptide sequence for all isoforms in FASTA format (FAA). These files are generated for each isoform by the Gene Model Checker and then concatenated by the Annotation Files Merger tool described below. They also submit an Annotation Report ^31^ to document the evidence supporting their proposed gene models.

***Exceptions to the standard annotation workflow***

While the standard annotation workflow provides a good starting point for the annotation of target genes in the Pathways Project, additional tools and strategies are needed to address challenges with the annotations of a subset of genes. Resolving these challenges typically require the integration of multiple lines of empirical and computational evidence. Below we describe the process for resolving two common challenges—non-canonical splice sites and assembly errors—and provide a list of other potential challenges annotators may encounter.

***Non-canonical splice sites***

The most common (canonical) sequence for the splice donor site is GT (GU in the pre-mRNA), and the most common sequence for the splice acceptor site is AG. Variant splice sites are termed non-canonical. For example, the GC splice donor site appears in ~0.8% (603/71,922) of the unique introns in *D. melanogaster* (FlyBase release 6.43). The use of a non-canonical splice site in a gene model will typically be supported by splice junction predictions derived from spliced RNA-Seq reads. The presence of a non-canonical splice site in the orthologous intron in the reference species ( *D. melanogaster*), or in multiple *Drosophila* species closely related to the target species, can also be used as supporting evidence for the annotation of a non-canonical splice site.

***Assembly errors***

Each sequencing platform (e.g., Sanger, Illumina, PacBio, and Nanopore) has a distinct error profile ^32^^,^^33^ that could introduce errors (e.g., base substitutions, insertions, and deletions) into the consensus sequence of an assembly. Transposons and other repetitive sequences (e.g., tandem repeats) in eukaryotic genomes can also lead to gaps and misassemblies. ^34^ Gene annotation challenges caused by assembly errors include partial or missing genes due to gaps in the assembly, apparent frameshifts within CDSs due to extra or missing nucleotides in the consensus sequence, and errors in ortholog/paralog assignments (e.g., due to “duplications” caused by misassemblies).

The publicly available genome assemblies utilized by the Pathways Project were constructed using different sequencing technologies and assembly protocols. Since these genome assemblies have not been manually improved, they might contain assembly errors that could interfere with coding region annotations. Consequently, in cases where proposed gene models for the target species includes changes in gene structure compared to the *D. melanogaster* ortholog (e.g., novel/missing isoforms, exons, and/or introns), further investigations are needed to ascertain if the difference is caused by an assembly error or reflects true divergence. As part of this assessment, the student evaluates multiple lines of evidence including: (1) sequence conservation with other *Drosophila* species besides *D. melanogaster*, (2) consistency with Illumina genomic reads and RNA-Seq reads in the NCBI Sequence Read Archive (SRA), and (3) consistency with other genome assemblies for the same species. ^35^

***Other exceptions***

Past studies have shown that the number of genes involved in the ISP varied in the different *Drosophila* species due to gene duplications and pseudogenization, ^36^ which leads to challenges in ortholog assignments. Other annotation challenges are caused by changes in gene structure (e.g., gain or loss of coding exons and isoforms) compared to the target gene in the reference species.

Another set of challenges pertain to gene models that do not conform to the typical characteristics of protein-coding genes, including genes with a non-canonical start codon (e.g., *Akt*), stop codon readthrough (e.g., *jim*), or trans-splicing (e.g., *mod (mdg4)*).

***Common issues in gene models prior to submission***

The Gene Model Checker sometimes reports multiple “fails” for a proposed gene model because it deviates from the expected biological characteristics of most protein-coding genes (e.g., gene model with multiple in-frame stop codons). Typically, the multiple failures can be attributed to an error in the upstream regions of the proposed gene model that propagates downstream.

For example, one common cause of multiple failures is frameshifts caused by selecting incompatible donor and acceptor splice sites. When a coding exon ends in an incomplete codon, the 3’ end of that coding exon and the 5’ beginning of the next coding exon must include nucleotides that form a complete codon once the intron has been spliced out. The number of nucleotides between the end of the last complete codon and the splice donor site is defined as the phase of the splice donor site. Similarly, the number of nucleotides between the splice acceptor site and the start of the first complete codon is defined as the phase of the splice acceptor site. In order to maintain the open reading frame (ORF) after the intron has been spliced out, the sum of the donor and acceptor phases for adjacent coding exons must either be zero (i.e., no extra codon) or three (i.e., one extra codon). Selecting incompatible donor and acceptor splice sites causes a frameshift that changes the reading frames of the coding exons downstream of that splice junction. Using the incorrect reading frame to translate the downstream coding exons will likely introduce stop codons in the translation, thereby triggering multiple failures in the Gene Model Checker.

To resolve gene models with multiple fails, the student starts troubleshooting at the beginning of the gene. In many cases, correcting errors in the upstream portion of the gene model resolves the fails reported downstream.

### Reconciliation

While most of the gene models produced by GEP students using the annotation protocol described above are congruent with each other, incongruent models require further examination by a student reconciler. Reconciliation is carried out by experienced students who have received additional training under the guidance of a GEP faculty, and/or senior-scientist, mentor.

Each target gene in a target species is annotated by at least two students working independently. This quality control step is predicated on the assumption that it is relatively common for one student to make a single error but relatively rare for multiple students working independently to make the same error.

Student reconcilers look for differences in the submitted gene models, paying special attention to the three most common errors that might invalidate a model (described below), and investigate any large-scale anomalies (e.g., proposed novel isoform and missing specific exons or isoforms).

**Reconciliation process**

**Figure 5.**
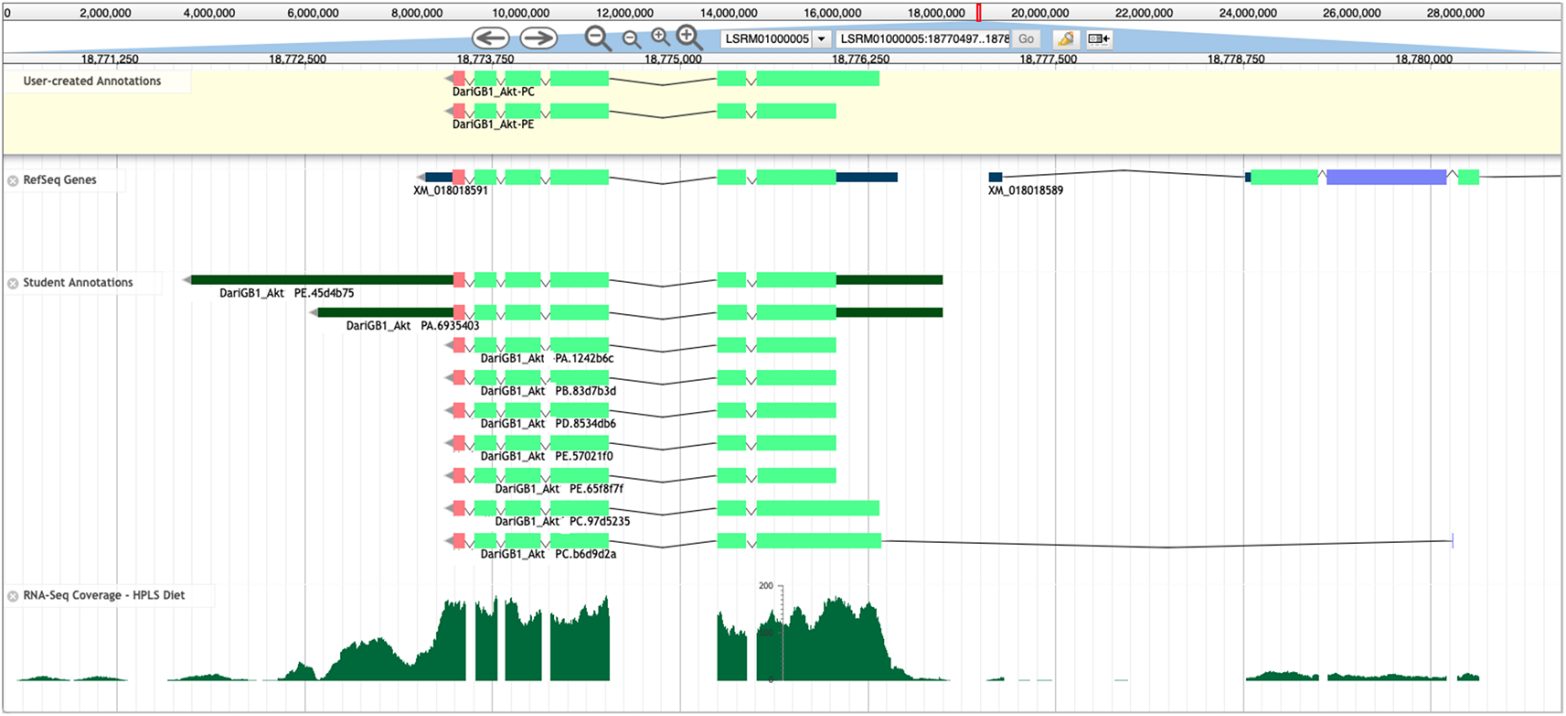
Apollo Screenshot for the *Akt* gene model in *D. arizonae.* Final gene models for *Akt* in *D. arizonae* (User-created Annotations track, yellow background), along with the NCBI RefSeq gene model (RefSeq Genes track), submitted student models (Student Annotations track), and RNA-Seq data aligning to the region (RNA-Seq Coverage – HPLS Diet track, histograms). Despite RefSeq predicting only one isoform (XM_018018591) for this gene, the final model contains two unique protein-coding isoforms (Akt-PC and Akt-PE), which were annotated using multiple lines of evidence. The Akt-PE isoform has a larger coding region in the reconciled gene model that is missed by the RefSeq gene predictions. In the Student Annotations track, the top two models included annotations of the 5’ and 3’ untranslated regions (UTRs, thinner dark green rectangles), and the bottom student model (DariGB1_Akt-PC.b6d9d2a) incorporated a coding exon that was likely not part of *Akt.*

Reconciliation is performed using Apollo, ^15^ a web-based collaborative genome annotation editor that allows reconcilers to view student-generated models alongside the evidence tracks ( Figure 5) used for annotating those models.

Reconcilers evaluate the available student annotations for each isoform in conjunction with the other evidence tracks (e.g., sequence similarity, RNA-Seq data, and gene predictions) to construct the final gene model for the protein-coding isoform(s) that is best supported by the available evidence. Reconcilers then draft a *microPublication* describing the supporting evidence for the final gene model. ^16^ The student annotators and their faculty mentors review and approve the article draft. Reconciled models are also used in downstream meta-analyses and deposited into the NCBI Third Party Annotation (TPA) database.

## Results

### CDS annotation procedure

**Identify the ortholog**

**Figure 6.**
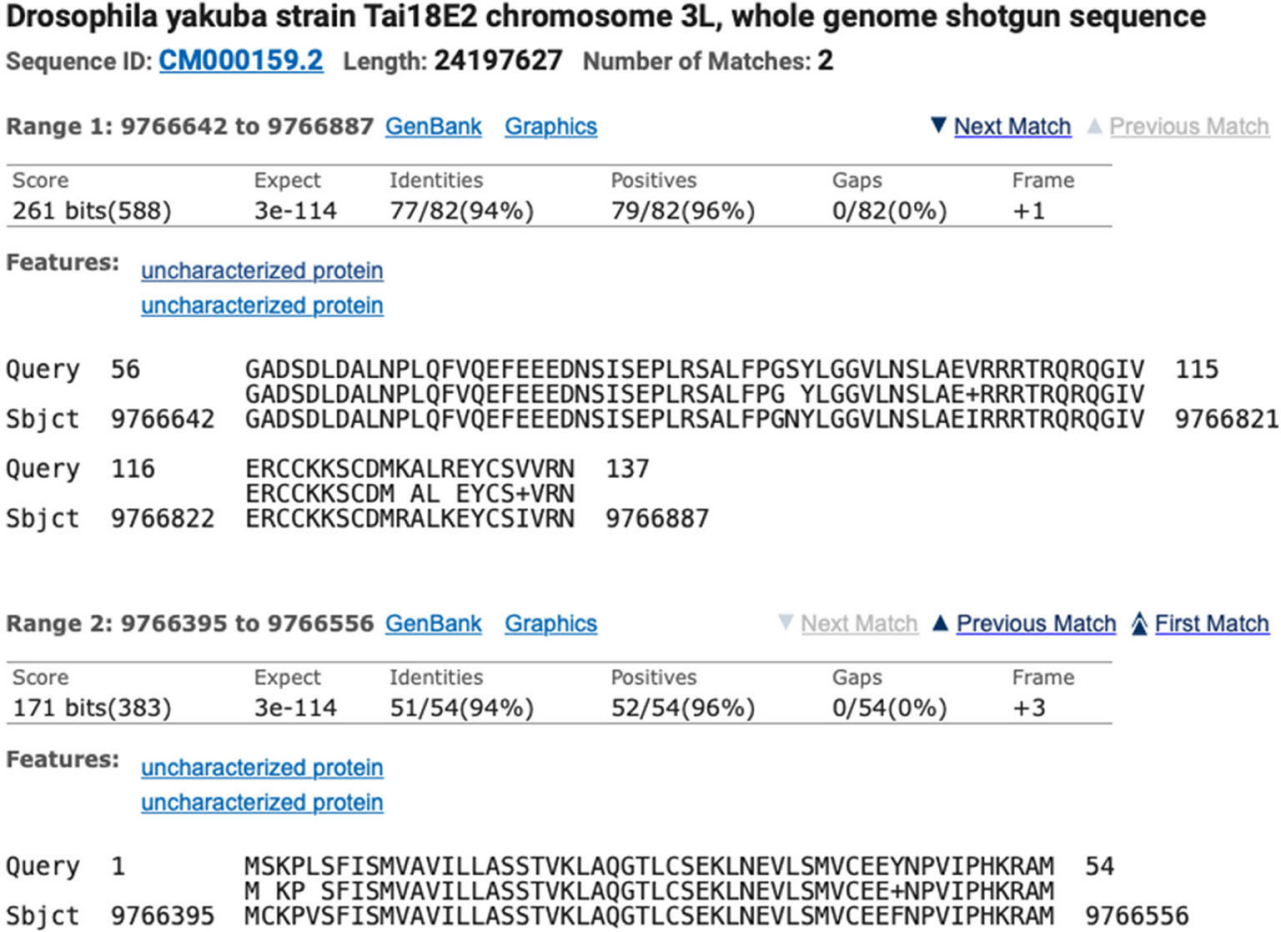
*tblastn* alignment for *Ilp2* from the *D. melanogaster* dm6 assembly (query) against the *D. yakuba* DyakCAF1 assembly (GCA_000005975.1; subject).

Identification of the ortholog is done using BLAST and local synteny analysis of the genomic neighborhood of the target gene. For example, to locate the *Ilp2* gene (target gene) in *D. yakuba* (target species), a *tblastn* search was used to compare the protein sequence for *D. melanogaster* Ilp2-PA (query) against the *D. yakuba* DyakCAF1 genome assembly (subject). The two collinear alignment blocks that correspond to the best match to the *D. melanogaster* Ilp2-PA protein (137aa) are located in the 9,766,395-9,766,887 region of the *D. yakuba* scaffold CM000159.2 (chr3L) ( Figure 6). The alignment block for the first 54aa of Ilp2-PA is located at 9,766,395-9,766,556 in frame +3, with a normalized score of 171 bits and 94% identity. The alignment block for residues 56-137 of Ilp2-PA is located at 9,766,642-9,766,887 in frame +1, with a normalized score of 261 bits and 94% identity. The joint E-value for the two collinear alignment blocks is 3e-114. The two alignment blocks account for 136aa out of 137aa of *D. melanogaster* Ilp2-PA (residue 55 of the protein is not covered by the two alignment blocks). Local synteny analysis shows that the genomic neighborhood surrounding *Ilp2* is conserved between *D. melanogaster* and *D. yakuba* ( Figure 3).

Collectively, the available evidence supports the hypothesis that the 9,766,395-9,766,887 region of the *D. yakuba* scaffold CM000159.2 contains the putative ortholog of *Ilp2.*

**Identify the coordinates of each coding exon**

The approximate coding exon coordinates for the target gene in the target species are defined by *tblastn* searches of the coding exons of the target gene in *D. melanogaster* against the genomic scaffold in the target species determined by the “Identify the ortholog” step. The approximate coding exon coordinates are then refined by examining the evidence tracks in the GEP UCSC Genome Browser. For example, Figure 4A shows the approximate placement of the second coding exon of the *Rheb* gene in the *D. yakuba* DyakCAF1 assembly based on the results of the *tblastn* search (i.e., scaffold CM000160.2 (chr3R) at 17,358,844-17,358,912 in frame +1). In Figure 4B, the refined start coordinate for the second coding exon of *Rheb* (i.e., at 17,358,842) was determined by RNA-Seq read coverage, splice junction predictions, gene predictions, and protein alignments evidence tracks on the GEP UCSC Genome Browser. Table 2 shows the refinement of all CDS exons of *Rheb* in *D. yakuba.* The “Difference” column indicates the necessity of manually refining BLAST derived coordinates.

**Verify gene model**

**Figure 7.**
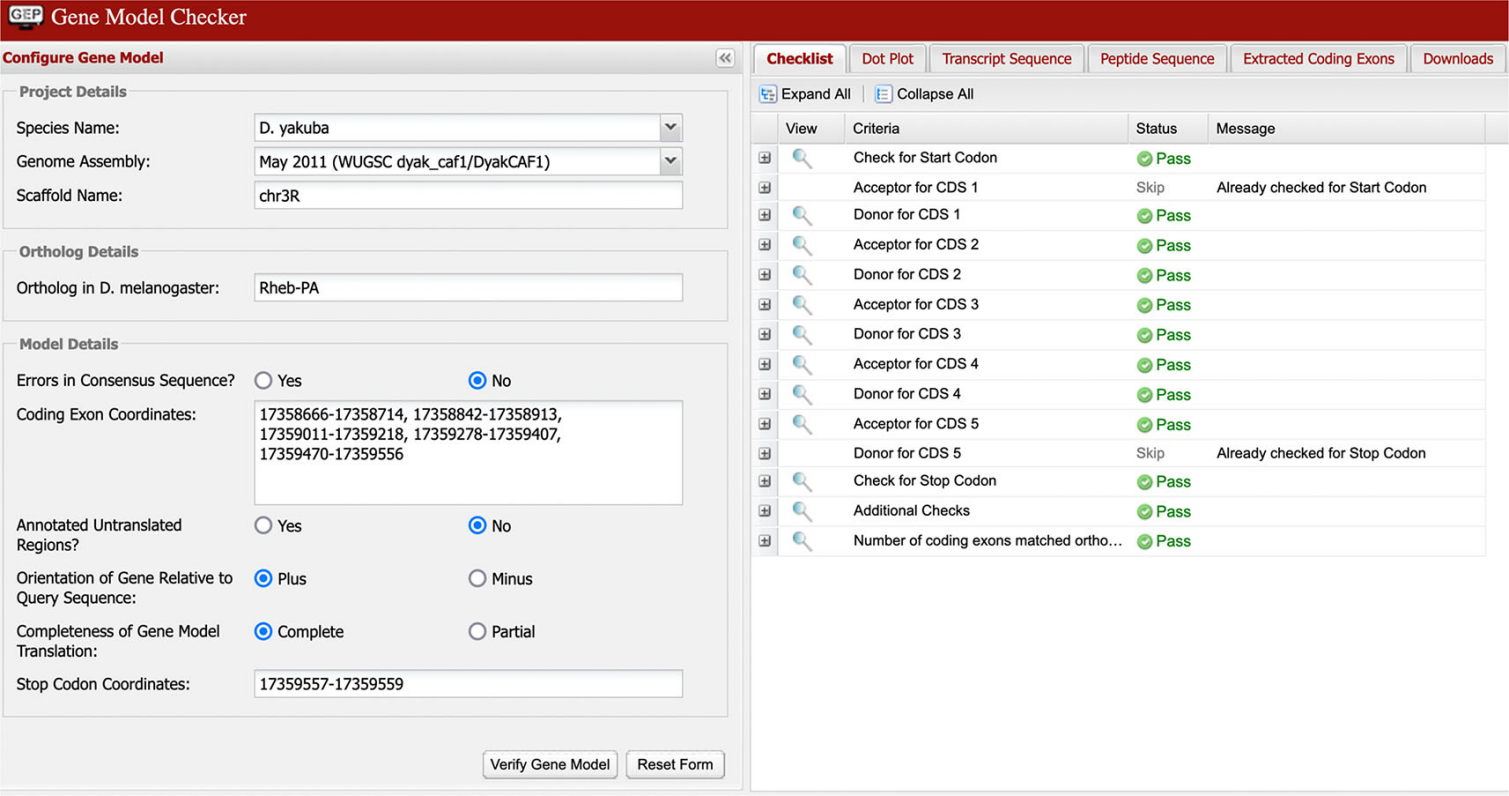
Confirmation that the coding exon coordinates of the putative ortholog of Rheb-PA in the *D. yakuba* DyakCAF1 assembly (GCA_000005975.1) follow molecular biology rules for protein-coding genes and match the expected number of exons in *D. melanogaster* using the GEP’s Gene Model Checker.

The final step prior to submission to the GEP is for the student to confirm the proposed gene model using GEP’s Gene Model Checker. This tool can help the annotator verify that the proposed gene model satisfies the biological constraints of most protein-coding genes and identify differences between the proposed gene model and the *D. melanogaster* ortholog. In Figure 7, we can see the checklist for the model of Rheb-PA in *D. yakuba* passed the Gene Model Checker (i.e., the proposed gene model begins with a start codon, ends with a stop codon, and the five coding exons use the canonical splice donor and acceptor sites). It is, however, possible to “pass”’ all the checks in the tool and still have an entirely incorrect model since the tool does not specifically test for protein sequence conservation or orthology.

**Most common annotation errors**

Reconciliation consists of reviewing two or more gene models created by student annotators working independently. The main advantage of manually curated gene models relative to computational predictions is the ability for the curator (in this case the student annotator) to evaluate and integrate across non-conforming pieces of evidence. Reconcilers provide further quality control measures as they closely scrutinize each idiosyncrasy in the gene models proposed by student annotators. The most common idiosyncrasies/errors are listed below.

***Selection of incorrect splice sites***

One of the most common annotation errors is the selection of splice donor and acceptor sites that are not the most parsimonious candidates compared to the target gene in the reference genome. Another type of splice site error is caused by the annotators placing too high of a priority on the use of canonical sequences for splice donor sites (GT) and splice acceptor sites (AG). While non-canonical splice sites are rare in *Drosophila* (i.e., found in < 1% of introns), they should be used if the non-canonical splice sites are supported by RNA-Seq data and conservation in other *Drosophila* species. ^37^^–^^39^

**Figure 8.**
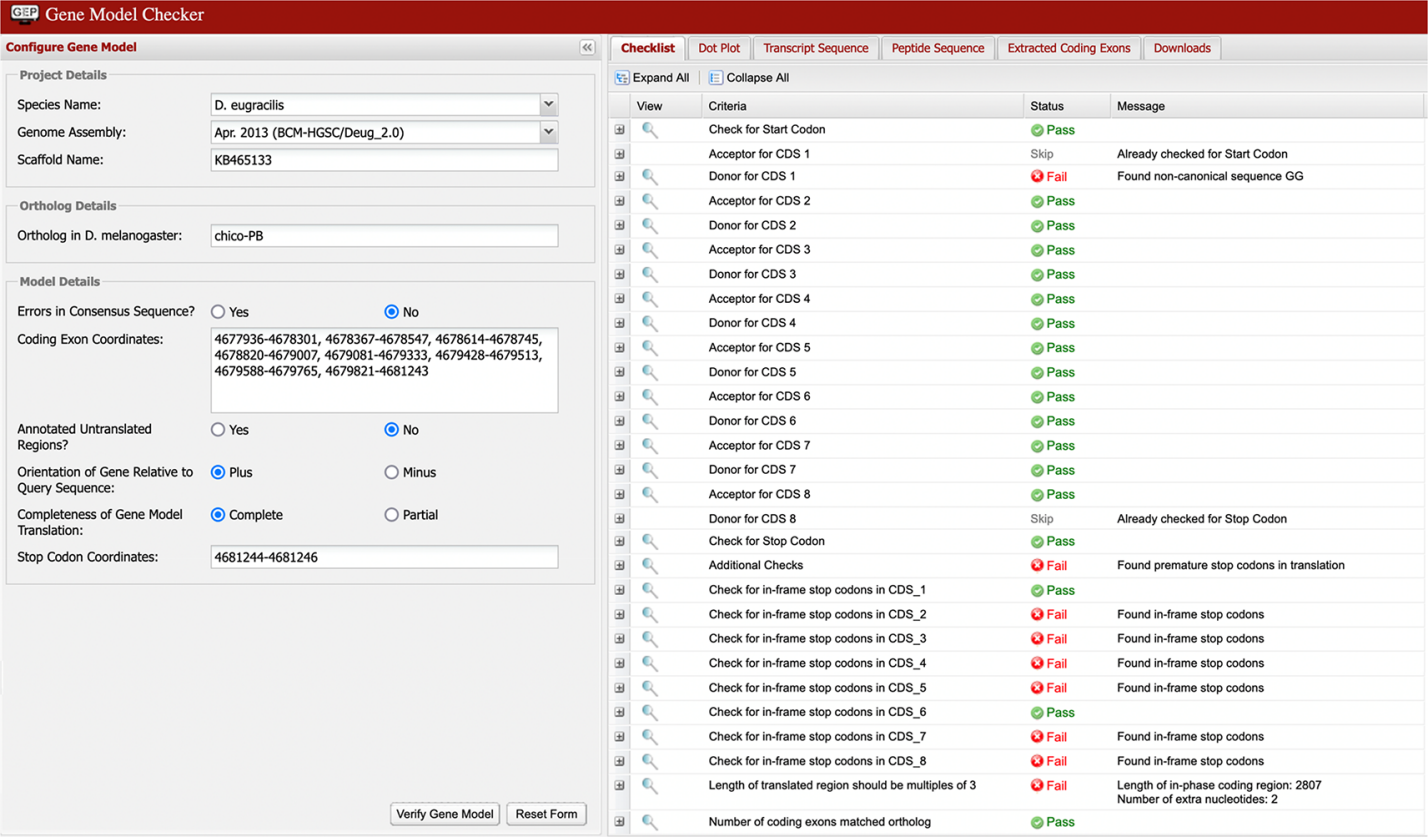
The Gene Model Checker checklist indicated the presence of in-frame stop codons within coding exons 2, 3, 4, 5, 7, and 8 of the proposed gene model for chico-PB in *D. eugracilis.* The checklist also reports the use of a non-canonical GG splice donor site for CDS 1, and that the total length of the coding region (2,807 nt) is not divisible by three. To address these errors, the annotator should verify the annotation for the item associated with the first error in the checklist (i.e., the end coordinate for the first coding exon and its corresponding splice donor site). In this example, the end of the first coding exon should be changed from 4,678,301 to 4,678,302 based on the available evidence on the GEP UCSC Genome Browser. This change will resolve the remaining failures in the checklist.

**Figure 9.**
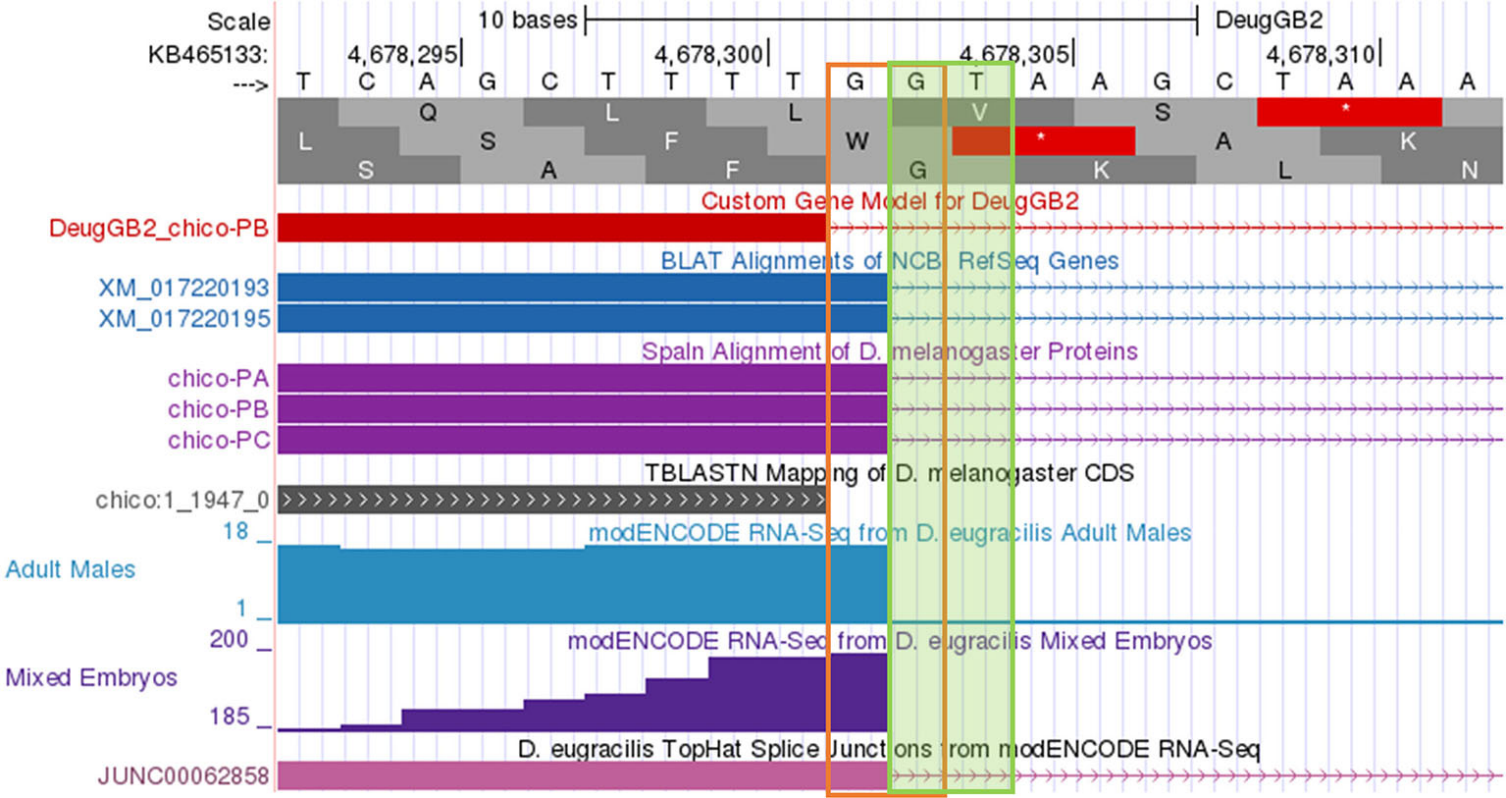
Use of incorrect splice site (GG) in the annotation of the *chico* ortholog in *D. eugracilis.* Extending the end of the proposed coding exon by one nucleotide (to 4,678,302) will utilize the canonical splice donor site (GT) and is more consistent with the available gene predictions and RNA-Seq data. The revised coding exon coordinate is supported by the splice junction prediction JUNC00062858 (score = 216).

Multiple failures in the Gene Model Checker checklist (as shown in Figure 8) are typically caused by the selection of incompatible splice sites during the “Refine coding exon coordinates” stage of the analysis. Most of these errors can be resolved by scrutiny of the coordinates for the checklist item where the first error is reported by the Gene Model Checker. Figure 9 shows the incorrect assignment of a splice site in *D. eugracilis* indicated by the first “fail” in the Gene Model Checker in Figure 8. The proposed splice donor site is one nucleotide away from the correct splice donor site with the canonical sequence of GT. Reconcilers are well equipped to identify the proper splice site when the student model has an error.

***Missing/extra exons***

Another common error is gene models with missing or extra coding exons compared to the *D. melanogaster* ortholog. Sometimes these proposed changes in gene structure are well supported by the data (e.g., a novel intron supported by splice junction predictions), but often the mismatch in the number of coding exons is due to the student annotator failing to fully account for all lines of evidence.

***Incorrect ortholog assignment***

Correct assignment of an ortholog depends on the proper configuration of the BLAST search parameters, proper interpretation of the BLAST search result, and proper consideration of local synteny compared to the reference species. If a student annotates a gene that is not the ortholog, then their gene model cannot be used to establish the final ortholog model in the target species.

### Exceptions to the standard annotation workflow

**Non-canonical splice sites**

**Figure 10.**
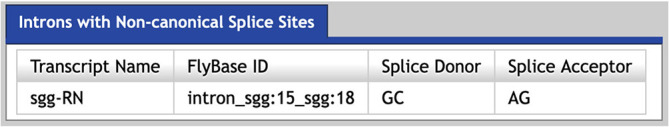
The “Introns with Non-canonical Splice Sites” section of the Gene Record Finder for the N isoform of *sgg* shows the presence of a non-canonical GC splice donor site in *D. melanogaster.*

While non-canonical splice sites are rare in *Drosophila* (i.e., found in < 1% of introns), they should be included if they are supported by RNA-Seq read coverage, splice junction predictions, and conservation in other species. ^37^^–^^39^ An example of a gene with a non-canonical spice site is sgg-RN in *D. melanogaster*, which has a GC splice donor site rather than the GT donor site between exons sgg:15 and sgg:18 ( Figure 10). The presence of a non-canonical splice site in a proposed model will be indicated by a “Warning” label in the Gene Model Checker. If a non-canonical splice site is substantiated by multiple lines of evidence (e.g., RNA-Seq data, conservation across multiple species), the student may conclude that the non-canonical splice site is also present in the target species.

**Assembly errors**

**Figure 11.**
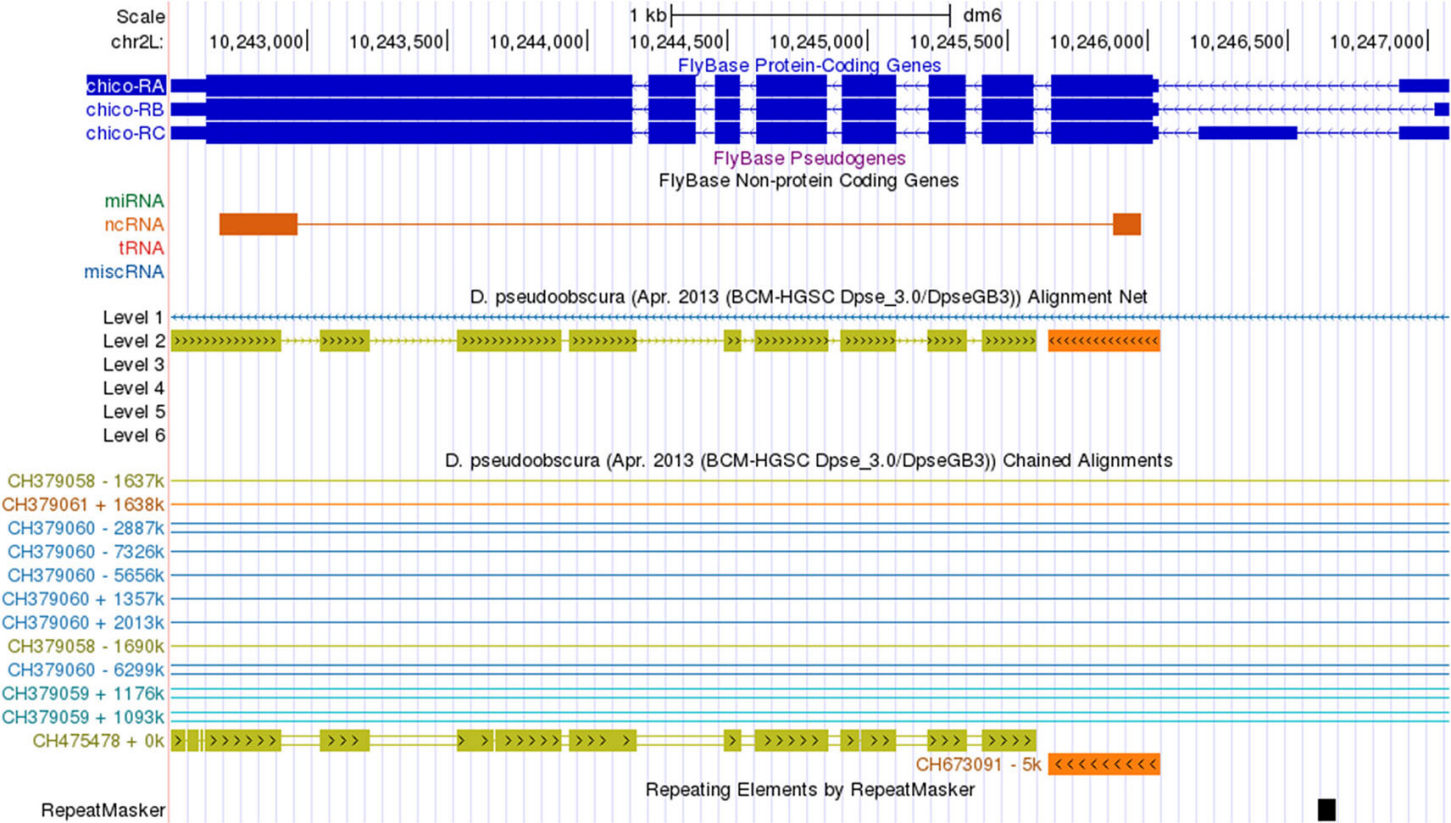
The Chained Alignments track in the *D. melanogaster* dm6 assembly shows that *chico* in the *D. pseudoobscura* DpseGB3 assembly (GCA_000001765.2) has been split across two scaffolds (CH475478 and CH673091).

Assembly errors (e.g., gaps) can affect the coding region gene annotations. The Chained Alignments between the *D. melanogaster* dm6 assembly and the *D. pseudoobscura* DpseGB3 assembly (GCA_000001765.2) show that the *chico* gene is split across two different scaffolds in the *D. pseudoobscura* DpseGB3 assembly—the first coding exon of *chico* is located in the *D. pseudoobscura* scaffold CH673091 and the rest of the gene is located in scaffold CH475478 ( Figure 11).

### Reconciliation

**Figure 12.**
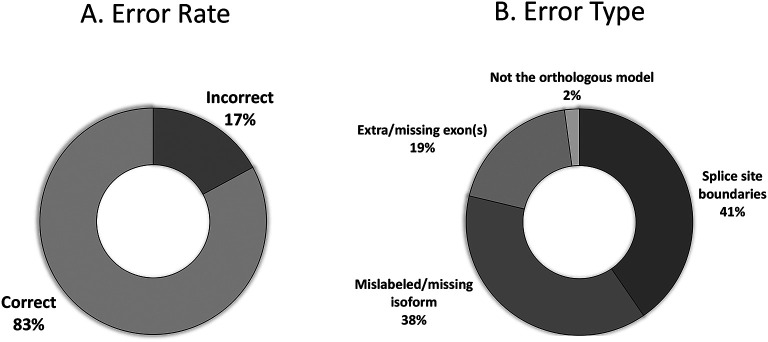
Error rate of student models that reach reconciliation (n=310). (A) The raw error rate of models, and the (B) breakdown of the error rates into the classes of errors.

The primary goal of reconciliation is to elucidate any inconsistencies or idiosyncrasies that might occur with two independent students generating the models. The majority of student models received for the Pathways Project (n=310) agree with the final reconciled model (83%; Figure 12A). Those with errors include mislabeled or missing isoforms (38%), incorrect splice sites (41%), extra or missing exon(s) (19%), and failing to identify the proper ortholog (2%) as seen in Figure 12B.

### microPublication

After student models are reconciled and manuscripts are drafted, they are sent for review and publication via the *microPublication* journal ( https://www.micropublication.org). The goal of this journal is to publish brief and novel findings whose results may lack a broader scientific narrative. The gene models generated and reconciled by students fit neatly within this description (e.g., Ref. 40).

## Discussion

This annotation protocol has been used by GEP faculty to engage undergraduate students in the comparative annotation of ISP genes in 27 *Drosophila* species. Similar protocols have previously been used by the GEP on other scientific projects. ^41^^–^^44^ Those protocols were used as the basis to develop the protocol described here.

To ensure gene models produced by GEP students are of high quality, each gene is annotated by at least two students working independently and then reconciled by a more experienced student. This manual curation process ensures the availability of an accurate set of gene models from multiple *Drosophila* species for the comparative study of network architecture and the evolution of genes involved in signaling pathways (e.g., the ISP).

The annotation effort of the student annotators is of value because the students can outperform computational gene predictors. For example, GEP students were able to annotate a conserved isoform not predicted by the NCBI RefSeq pipeline for *Akt* in *D. arizonae* ( Figure 5). This isoform was not predicted computationally due to a non-canonical ACG start codon that is conserved in *Akt* in *D. melanogaster.*

Annotation and reconciliation protocols similar to the one described in this article are also currently used to investigate the expansion of the Muller F element in four *Drosophila* species and the evolution of venom proteins in four parasitoid wasp species. Faculty can also use this protocol to create new Course-based Undergraduate Research Experiences (CUREs) that engage students in the comparative analysis of genes involved in other metabolic and signaling pathways.

The protocol described here is focused only on the annotation of coding regions. Once additional experimental data (e.g., CAGE, RAMPAGE, and long-read RNA-Seq data) becomes available in more species, future analyses will focus on the annotation of untranslated regions and transcription start sites.

## Data and software availability

### Repository-hosted data

**Repository: NCBI**

The GenBank accession numbers for the assemblies and BioProject accession numbers for the RNA-Seq datasets are available on NCBI under the accession numbers listed in Table 1. Data is available in the public domain through NCBI ( https://www.ncbi.nlm.nih.gov).

**Repository: FlyBase**

FlyBase data used for tool creation mentioned below is publicly available through the FlyBase FTP site ( https://ftp.flybase.net/releases/).

### Data that cannot be shared

**Data under license by a third party**

The UCSC Genome Browser is developed by the Genomics Institute at the University of California Santa Cruz. The source code for the UCSC Genome Browser is covered by five different licenses. Most of the source code is available under the MIT License, and all the source code is freely available to non-commercial entities. The source code is available on GitHub ( https://github.com/ucscGenomeBrowser/kent), and it can also be obtained through the UCSC Genome Browser Store ( https://genome-store.ucsc.edu/).

**Large data**

The datasets displayed in the GEP UCSC Genome Browser are too large to be feasibly hosted by an F1000Research-approved repository, such as RNA-Seq read alignments and Whole-Genome Multiple Sequence Alignments. The data is available through the GEP UCSC Genome Browser and the UCSC Table Browser ( https://gander.wustl.edu). Details on the datasets and tools used to construct each evidence track are available through the settings page for each track in the GEP UCSC Genome Browser. As stated under the “Repository-hosted Data” section, the genome assemblies and the RNA-Seq datasets used to construct the evidence tracks on the GEP UCSC Genome Browser are in the public domain, and they can be obtained through NCBI ( https://www.ncbi.nlm.nih.gov).

### Software

Repository: GitHub

• Annotation Files Merger ( https://github.com/wilsonleung-gep/annotation-files-merger)
• Dot Plot Viewer ( https://github.com/wilsonleung-gep/dot-plot-viewer)
• Gene Record Finder ( https://github.com/wilsonleung-gep/gene-record-finder)
• Sequence Updater ( https://github.com/wilsonleung-gep/sequence-updater)
• Small Exons Finder ( https://github.com/wilsonleung-gep/small-exons-finder)

All the source code in the above repositories is available under the MIT License.

• Gene Model Checker ( https://github.com/wilsonleung-gep/gene-model-checker)
  The Gene Model Checker is available under the GNU General Public License v3.0.

All custom software and tools generated by the GEP can be accessed from the GEP website ( https://thegep.org). The annotation tools are publicly available web-based applications, thus requiring no installation on the part of the user. Versions of the tools can be found in Table 3. All the GEP annotation tools are synchronized to the same FlyBase release. The FlyBase *D. melanogaster* gene annotations used by the GEP annotation tools are updated twice a year (i.e., just before the start of the Fall and the Spring semesters) in order to mitigate the impact of FlyBase updates to the *D. melanogaster* reference gene annotations during the semester.

**Table 3.** Software Version Information and release dates for the web-based applications used by the manual gene annotation protocol for the Pathways Project.

Software	Version	Release Date
GEP UCSC Genome Browser	v400; FlyBase 6.43	Dec. 31, 2021
Gene Model Checker	v2.0; FlyBase 6.43	Dec. 31, 2021
Sequence Updater	v2.0	Jan. 15, 2021
BEDTools	v2.30.0	Jan. 23, 2021
Gene Record Finder	v1.3; FlyBase 6.43	Dec. 31, 2021
Small Exons Finder	Prototype; v1.0	Dec. 31, 2020
Annotation Files Merger	v2.0; FlyBase 6.43	Dec. 31, 2021
BLAST+	v2.12.0	June 28, 2021

**GEP UCSC Genome Browser**

Since GEP materials are catered towards undergraduates that may have limited or no experience with programming and command-line interfaces, we endeavor to make all data easily accessible, thereby reducing the barrier to engagement in genomics research. The GEP maintains a mirror of the UCSC Genome Browser with *Drosophila* genomes ( https://gander.wustl.edu). We created these Genome Browsers ^45^ with multiple evidence tracks using data generated by various algorithms ^4^ and experimental data obtained from the NCBI SRA.

**Gene Record Finder**

The Gene Record Finder ( https://gander.wustl.edu/%7ewilson/dmelgenerecord/index.html) summarizes the FlyBase annotations for protein-coding genes in *D. melanogaster.* It provides information about the structure of each *D. melanogaster* gene, such as the number of isoforms (and number of isoforms with unique coding regions), as well as the amino acid sequence for each coding exon and the nucleotide sequence for each exon. The Gene Record Finder also provides exon usage maps that demarcate the exons used by each isoform. This information is used in the “Identify the approximate coordinates of each coding exon” step of the annotation protocol. While this information can be directly obtained from FlyBase, annotators can more easily retrieve the gene structure information from a single page instead of through multiple FlyBase Transcript and Polypeptide Reports.

**Gene Model Checker**

The Gene Model Checker ( https://gander.wustl.edu/%7ewilson/genechecker/index.html) provides a way for annotators to check their own work when constructing gene models. It verifies that the proposed gene model satisfies basic biological constraints (e.g., maintains an ORF, uses canonical start and stop codons, and canonical splice donor and acceptor sites). It also indicates whether the number of coding exons for the proposed gene model in the target species matches that in the orthologous isoform in *D. melanogaster.* If there is empirical evidence indicating the gene has unusual characteristics (e.g., the use of a non-canonical start codon, stop codon read-through, or polycistronic transcripts ^18^), students provide the supporting evidence for their claims in the Annotation Report form.

The “Dot Plot” section of the Gene Model Checker output compares the proposed gene model against the putative ortholog in *D. melanogaster* using protein alignment algorithms. Large gaps in the dot plot or protein alignment might indicate the selection of an incorrect splice site, missing exons, or extra exons in the proposed gene model. Note that this tool does not compare the proposed gene model against other lines of evidence, such as RNA-Seq data or computational gene predictions. The Gene Model Checker also produces the three annotation files for the proposed gene model that are required for project submission (a GTF file to define genomic feature coordinates, a transcript sequence file in FASTA format (FNA), and a peptide sequence file in FASTA format (FAA)).

The Gene Model Checker requires input of the species, assembly, and the scaffold of the putative ortholog. In addition, the Gene Model Checker requires the name of the ortholog in *D. melanogaster,* the set of coding exon coordinates for the gene in the target species, orientation of the gene, noting whether or not the untranslated regions are included, whether the gene model is complete (i.e., encompasses all CDSs/UTRs), and whether the genomic region containing the gene in the target species has consensus errors (e.g., Figure 7). Presently, this tool only supports the 28 *Drosophila* species that are currently in the GEP UCSC Genome Browser.

**Small Exons Finder**

The typical use case for the Small Exons Finder ( https://gander.wustl.edu/%7ewilson/smallexonfinder/index.html) tool is identifying CDSs that are too small or too weakly conserved to be detected by BLAST. The tool is designed to look for ORFs that satisfy a set of biological constraints including the type of CDS (i.e., initial, internal, or terminal CDS), the phase of the donor or acceptor splice site, and the expected CDS size according to the *D. melanogaster* model. The Small Exons Finder then looks for ORFs in the provided sequence that conform to the aforementioned constraints. Compared to the ORF-FINDER tool developed by NCBI, ^46^ the Small Exons Finder allows users to search for ORFs that are less than 30bp in size and can search for initial, internal, and terminal coding exons with constraints on the phases of the donor and acceptor sites.

**Sequence Updater**

The Sequence Updater ( https://gander.wustl.edu/%7ewilson/sequence_updater/index.html) tool is primarily used to create a Variant Call Format (VCF) file ^47^ to correct errors (i.e., base substitutions, insertions, and deletions) in the consensus sequence of a genome assembly. The VCF file produced by the Sequence Updater can be used with the Gene Model Checker to validate a gene model with consensus errors.

**Annotation Files Merger**

The Gene Model Checker produces GFF, FNA, and FAA files for each isoform. The submission pipeline requires a single GFF, FNA, and FAA file that includes all the unique isoforms in a project. For each type of annotation file, the Annotation Files Merger ( https://gander.wustl.edu/%7ewilson/submissionhelper/index.php) is used to combine the annotations for all the unique isoforms in a project into a single file. The Annotation Files Merger also enables the user to view the combined GFF file as a custom track on the GEP UCSC Genome Browser.

### Third party tools

**BEDTools**

BEDTools ^48^ was used to perform genomic arithmetic and compare locations of genomic features in multiple tracks of the GEP UCSC Genome Browser.

**NCBI BLAST+**

The local sequence alignments produced by the tools in NCBI BLAST+ suite were used for multiple aspects of the protocol including, but not limited to, sequence annotation and local synteny assignment.

### Extended data

Figshare. Supplement 1.pdf, DOI: https://doi.org/10.6084/m9.figshare.21235341

Figshare. Supplement 2.pdf, DOI: https://doi.org/10.6084/m9.figshare.21235345

Figshare. Supplement 3.docx, DOI: https://doi.org/10.6084/m9.figshare.21235376

Figshare. Supplement 4.docx, DOI: https://doi.org/10.6084/m9.figshare.21235367

Figshare. Supplement 5.pdf, DOI: https://doi.org/10.6084/m9.figshare.21235343

Figshare. Supplement 6.docx, DOI: https://doi.org/10.6084/m9.figshare.21235380

Data are available under the terms of the Creative Commons Zero “No rights reserved” data waiver (CC0 1.0 Public domain dedication).
