# Supplementary material for: Manual annotation of Drosophila genes: a Genomics Education Partnership protocol
Source: F1000Res. 2023 Oct 13;11:1579. Originally published 2022 Dec 23. [Version 3] doi: 10.12688/f1000research.126839.3 (PMC10579860; doi:10.12688/f1000research.126839.3)
Supplement: Peer review history

# Reviewer report: Reviewer response for version 3

**DOI:** 10.5256/f1000research.156420.r215145

**Published:** 2023-10-16

**Authors:** Terence D. Murphy

**Affiliations:** 1 National Center for Biotechnology Information, National Library of Medicine, National Institutes of Health, Bethesda, MD, USA

Thank you for the revisions to Figure 1. They serve to more accurately represent the differences between the gene prediction algorithms.

Is the rationale for developing the new method (or application) clearly explained?

Yes

Is the description of the method technically sound?

Yes

Are the conclusions about the method and its performance adequately supported by the findings presented in the article?

Yes

If any results are presented, are all the source data underlying the results available to ensure full reproducibility?

Yes

Are sufficient details provided to allow replication of the method development and its use by others?

Yes

Reviewer Expertise:

Expertise in manual and automated gene annotation techniques and supporting datasets

I confirm that I have read this submission and believe that I have an appropriate level of expertise to confirm that it is of an acceptable scientific standard.

# Reviewer report: Reviewer response for version 2

**DOI:** 10.5256/f1000research.147335.r192290

**Published:** 2023-09-07

**Authors:** Terence D. Murphy

**Affiliations:** 1 National Center for Biotechnology Information, National Library of Medicine, National Institutes of Health, Bethesda, MD, USA

Thank you for the revisions to the manuscript. I particularly appreciate the data on curation error rates (figure 13), and look forward to the TrackHub being available in the TrackHub Registry.

 I have three revisions, the first of which is significant:

 1) Figure 1 is still inaccurate and does need to be fixed. The authors make the assertion that "The number of predicted **genes** can show large variations across algorithms", stated in the figure title, legend, Y axis, and text. The legend goes on to explain that some algorithms "can predict multiple transcripts per genomic region (e.g., genBlastG, Spaln)". Having multiple transcripts/isoforms per gene should only be interpreted as inflating the protein-coding **gene** count if the algorithm does not provide information to group them into genes. And in fact the authors are applying such grouping for D. melanogaster by showing a baseline of ~14k genes, as opposed to the ~31k isoforms annotated by FlyBase. NCBI RefSeq provides information to group isoforms into genes, with 14181+/-1083 protein-coding genes reported in current Drosophila annotations. See: https://www.ncbi.nlm.nih.gov/datasets/genome/?taxon=7215&annotated_only=true&refseq_annotation=true

 And use "Select columns" to add the protein-coding gene count.

 While this isn't a critical point of the paper, the figure implies many of these algorithms are predicting twice as many protein-coding **genes** as found in D. melanogaster, which isn't true in most cases.

 a) The best fix is to correct the figure to show true counts of protein-coding genes, aggregating multiple isoforms where that information is provided by the algorithm. Then it's correct to compare to the 14k protein-coding genes annotated in D. melanogaster

 b) Alternatively, the authors can revise the main text, figure legend title and text, Y axis, and legend in the figure itself to state everything is plotted for isoforms, and revise the D. melanogaster baseline to be 30799 (for Release 6.46). That alternative isn't ideal, but would be less misleading.

 2) if the location will be reasonably permanent, please provide a URL for the TrackHub in the paper so that they can be accessed while the TrackHub Registry is pending.

 3) the "Missing/Mislabeled Isoforms" heading is formatted in a different style

Is the rationale for developing the new method (or application) clearly explained?

Yes

Is the description of the method technically sound?

Yes

Are the conclusions about the method and its performance adequately supported by the findings presented in the article?

Yes

If any results are presented, are all the source data underlying the results available to ensure full reproducibility?

Yes

Are sufficient details provided to allow replication of the method development and its use by others?

Yes

Reviewer Expertise:

Expertise in manual and automated gene annotation techniques and supporting datasets

I confirm that I have read this submission and believe that I have an appropriate level of expertise to confirm that it is of an acceptable scientific standard, however I have significant reservations, as outlined above.

# Reviewer report: Reviewer response for version 2

**DOI:** 10.5256/f1000research.147335.r192289

**Published:** 2023-08-24

**Authors:** Madeline Crosby

**Affiliations:** 1 Department of Molecular and Cellular Biology, Harvard University, Cambridge, MA, USA

Accepted as revised.

Is the rationale for developing the new method (or application) clearly explained?

Yes

Is the description of the method technically sound?

Yes

Are the conclusions about the method and its performance adequately supported by the findings presented in the article?

Partly

If any results are presented, are all the source data underlying the results available to ensure full reproducibility?

Yes

Are sufficient details provided to allow replication of the method development and its use by others?

Yes

Reviewer Expertise:

Relevant area of expertise: gene model annotation in Drosophila melanogaster.

I confirm that I have read this submission and believe that I have an appropriate level of expertise to confirm that it is of an acceptable scientific standard.

# Reviewer report: Reviewer response for version 1

**DOI:** 10.5256/f1000research.139289.r162520

**Published:** 2023-02-16

**Authors:** Madeline Crosby

**Affiliations:** 1 Department of Molecular and Cellular Biology, Harvard University, Cambridge, MA, USA

This paper is a detailed description of a protocol used for undergraduate-based annotation of gene models in various Drosophila species. Undergraduate students benefit from a hands-on experience using computational tools and real genomic data. As primarily a methods paper, it appears to be admirably thorough.  

 Two minor errors that need to be corrected:

1. Figure 5 legend confuses Akt-PC and Akt-PE.
2. Link for reference 40 goes to a retracted paper.

Additional comments, questions, and suggestions:

 The initial project-claiming step includes an estimated difficulty level; this is potentially very useful, but the current assessment appears to be based on the overly simplistic criteria of the number of isoforms, number of coding exons, and evolutionary distance from the reference genome. If this difficulty assessment could be expanded to include atypical phenomena such as non-canonical splicing and non-canonical start codons, it would be much more useful.

 Time permitting, it would be beneficial for a student to receive two genes to annotate: one simple case and a second more complicated case.

 Were students asked to complete the gene model for the described case of an incomplete genome assembly (split scaffold, Figure 11)?  I should think these would fall in the very difficult category and should be screened out.

 No ortholog identified by tBLASTn: Can you give an example of successful annotation of a model that was located based on synteny and pieced together from ‘additional features’ in the syntenic region? 

 “Correct” naming of protein isoforms is mentioned. The policy appears to be that an isoform should be named with the same suffix as the analogous isoform for the orthologous D. melanogaster gene. This is perhaps helpful, but it is not strictly necessary.

 Results using a group of 310 students who submitted gene models are very briefly described (Figure 12); it would be informative if this assessment were expanded. For example:

1. How effective was the strategy to have two independent students annotate a given gene? (Was an incorrect submission usually accompanied by a correct submission?)
2. What percentage of incorrect submissions were in the more difficult category?
3. What percentage of incorrect submissions involved atypical phenomena?

What percentage of students were unable to create a gene model on their own (initially)?  What type of assistance was provided to these students?

 Has feedback from students precipitated any changes?

Is the rationale for developing the new method (or application) clearly explained?

Yes

Is the description of the method technically sound?

Yes

Are the conclusions about the method and its performance adequately supported by the findings presented in the article?

Partly

If any results are presented, are all the source data underlying the results available to ensure full reproducibility?

Yes

Are sufficient details provided to allow replication of the method development and its use by others?

Yes

Reviewer Expertise:

Relevant area of expertise: gene model annotation in Drosophila melanogaster.

I confirm that I have read this submission and believe that I have an appropriate level of expertise to confirm that it is of an acceptable scientific standard, however I have significant reservations, as outlined above.

# Response

**Published:** 2023-06-27

**Authors:** Chinmay Rele

Thank you for your time and detailed descriptions of the ways you think our methods article could be better improved. We have made most of the changes that you have suggested, and have detailed them below.

- *Two minor errors that need to be corrected:*
  1. *Figure 5 legend confuses Akt-PC and Akt-PE.*
  2. *Link for reference 40 goes to a retracted paper.*

**The two minor corrections that have been suggested have been corrected. We have made these corrections pertaining to the Figure 5 legend confusing Akt-PC and Akt-PE , and also updated reference 40 to the correct final publication (note the initial retraction and republication was for a technical issue in the publication process, not a scientific issue)** .

- *The initial project-claiming step includes an estimated difficulty level; this is potentially very useful, but the current assessment appears to be based on the overly simplistic criteria of the number of isoforms, number of coding exons, and evolutionary distance from the reference genome. If this difficulty assessment could be expanded to include atypical phenomena such as non-canonical splicing and non-canonical start codons, it would be much more useful.*

**This is a very good suggestion, and we will definitely consider incorporating this kind of information in our claim pipeline in the future. The primary intent for including the difficult score is for faculty to gauge the approximate challenge of the gene to determine if it might be appropriate to their class, and is not a piece of information intended to be shared with the student. For this purpose, we have found that the current definition of the difficulty (including the number of polypeptides, isoforms, and species divergence) seems to be adequate. From the student perspective, the labor required to complete their project generally scales with the number of unique polypeptides and exons.** .

- *Time permitting, it would be beneficial for a student to receive two genes to annotate: one simple case and a second more complicated case.*

**It would definitely be beneficial to have students annotate (at least) two genes, one simple, and the second more complicated. We recommend to our faculty that they do this if possible, but, this decision ultimately lies with the faculty member who is implementing GEP curriculum within their course, and their pedagogical goals. Further, faculty are welcome to perform their own screening on the various potential projects before claiming them, to determine whether they feel a model is at the right scale for their students.**

- *Were students asked to complete the gene model for the described case of an incomplete genome assembly (split scaffold, Figure 11)? I should think these would fall in the very difficult category and should be screened out.*

**Instances where the assembly is idiosyncratic are prime examples where manual annotation of models by students is beneficial. Cases this extreme are rare. However, these kinds of cases are what make students most excited and proud of their work when they solve them. We do not recommend that a student’s first project be this difficult but once they understand the basics, many students are able to tackle these more complex scenarios. We also provide support to the faculty and students through virtual office hours and a Slack team with the project leaders for the faculty, virtual TAs for the students. We describe and analyze the effectiveness of our pedagogical approaches in other manuscripts either published and or in preparation. We have added some of those citations to the manuscript discussion.**

- *No ortholog identified by tBLASTn: Can you give an example of successful annotation of a model that was located based on synteny and pieced together from ‘additional features’ in the syntenic region?*

**Thank you for pointing out that we did not include an example. We apologize for this oversight. We have added an explanation of how to resolve an error like this by citing *Ilp3* in *D. grimshawi*. We have also added a figure to show this**

- *“Correct” naming of protein isoforms is mentioned. The policy appears to be that an isoform should be named with the same suffix as the analogous isoform for the orthologous D. melanogaster gene. This is perhaps helpful, but it is not strictly necessary.*

**Correct naming of protein isoforms is mentioned in order to maintain consistency across the entire project. Though this is not explicitly necessary, it is beneficial to assess and predict putative function of each transcript in the target species.**

- *Results using a group of 310 students who submitted gene models are very briefly described (Figure 12); it would be informative if this assessment were expanded. For example:*
  - *How effective was the strategy to have two independent students annotate a given gene? (Was an incorrect submission usually accompanied by a correct submission?)*
  - *What percentage of incorrect submissions were in the more difficult category?*
  - *What percentage of incorrect submissions involved atypical phenomena?*

**In general, we do see more errors in models classified as being more difficult, but understanding a more discrete error profile is out of the scope of this paper. The primary goal of this manuscript is to provide the description of the standard protocol used by the student annotators part of the GEP to annotate a gene within the target species. Once more data is available, we can make a more detailed assessment of the quality of student models relative to the nature of the gene characteristics.**

- *What percentage of students were unable to create a gene model on their own (initially)? What type of assistance was provided to these students?*

**We really cannot quantify what percentage of students were unable to create their own model initially for a variety of technical and semantic reasons, primarily since the reconciliation process only sees the summative product of the student work, not the formative steps the students experienced. We do know however that the formative frustration of the challenges of generating a model, followed by ultimate success is essential for driving the positive impact of the research experience (Lopatto et al., 2020; PMID: 32148609). The GEP provides a variety of support for students and their faculty mentors including extensive curriculum resources, video tutorials, virtual teaching assistants, and virtual office hours with project leaders.**

- *Has feedback from students precipitated any changes?*

**Student feedback is constantly considered as the curriculum and project protocols evolves to keep pace with changes in genomic data resources, with semi-annual updates. Student comments, especially in the pilot stages for new curriculum, are actively solicited by the project leaders. Further, the faculty and virtual TAs observations of how students may struggle with new concepts, inspire new or improved educational resources.**

 Thank you again for all your input to help us make this manuscript more complete and robust.

# Reviewer report: Reviewer response for version 1

**DOI:** 10.5256/f1000research.139289.r158890

**Published:** 2023-01-25

**Authors:** Terence D. Murphy

**Affiliations:** 1 National Center for Biotechnology Information, National Library of Medicine, National Institutes of Health, Bethesda, MD, USA

This paper describes a protocol for manual annotation of genes involved in insulin signaling pathway (ISP) in Drosophila species using a platform developed by Genome Education Partnership (GEP) as a tool to educate undergraduate students and get them interested in scientific research. The platform also helps generate valuable manually curated annotation in these species where there isn’t much evidence data available, by leveraging data from D. melanogaster. The protocol is well conceived to meet the needs of an educational program. The paper is generally sound, but would benefit from a few clarifications and corrections as described below.

  

1. While this is a ‘Methods” paper, the authors should perhaps provide some context in the Introduction about what GEP is and what its broader goals are (citing the latest publication on GEP) for the benefit of the naïve reader before diving into the details of the manual annotation protocol.
2. In figure 1, the Y-axis is labeled as “number of predicted protein-coding genes”, but the legend includes the following statement that implies it’s actually number of isoforms: “ *Prediction differences can partly be attributed to some algorithms predicting a single isoform in a genomic region (e.g., GeneID), while others predict multiple isoforms per genomic region (e.g., genBlastG, Spaln)*.”  Furthermore, the values for NCBI RefSeq Genes are all in the 20-45k range, when NCBI reports most Drosophila species as having 13-15k protein-coding genes. The authors need to check the data for all annotation programs provided in figure 1 and correct the figure.
3. In table 1, the authors list “UCSC Assembly” with names that largely aren’t shown in UCSC and don’t correspond to the published assembly names. For example, the D. miranda assembly GCA_000269505.2 is named DroMir_2.2. Where did the name DmirGB2 come from? The authors should clarify the meaning of the first column, and add an additional column with the official name for each assembly since it’s difficult to recognize assembly accessions.
4. Many of the assemblies being used by the GEP project have been superseded by newer and higher quality assemblies. Do the authors periodically upgrade to new assemblies, or is this viewed as unnecessary effort for the educational goals of the project?
5. To support the statement *“As of August 2022, GEP students from 79 institutions have used this annotation protocol to construct 2,101 gene models across 27 Drosophila species.”*, it would be useful to provide a supplemental table with the list of gene models curated for this project so far. Additional data on conformance of student models to the final consolidated annotations would also be of interest if readily available.
6. In order to aid access to the final gene models, in addition to the TPA submissions the authors could construct a genome browser track available through the remote Track Hub system. This could be updated periodically (e.g. at the end of each semester or year) with additional gene models, and made available through the Track Hub Registry system. While it would be useful to include the availability of this track in the paper if the authors have the resources to generate it, it’s not a requirement for publication.
7. Figure 5: It seems that the isoform names are reversed in the legend. Looking at the ‘User-created Annotations’ track, Akt-PC is the isoform with the longer coding region and not Akt-PE as stated in the legend.
8. Assembly errors: For cases where the student identifiers an error in a target assembly, how is that information conveyed in the TPA records? Is the TPA sequence corrected via a VCF file? What is done with a case like D. pseudoobscura chico from Figure 11?
9. For the reconciliation process, how would you determine that an isoform is ‘mislabeled’? Is it in comparison to D. melanogaster isoform name? In general, what are the rules to name isoforms in target species?
10. “ *The annotation effort of the student annotators is of value because the students can outperform computational gene predictors*.” - This statement seems a bit misleading unless there is a mention of scale. Computational annotation methods can predict gene and transcript models on a genomic scale, which is not possible by manual annotators (in a reasonable timeframe). It would be reasonable to say that manual annotators can correct errors found in computational predictions and can fill in gaps, as in the example cited in the paper where computational methods could not detect an additional protein isoform due to the use of an upstream non-AUG start codon. If the authors wish to keep the statement, a suggestion is to add something that conveys “at the level of an individual gene or a small set of genes” at the end. It would also be helpful to add some statistics on how often students identify errors or additions to automated gene sets.
11. The report is primarily focused on describing aspects of the annotation protocol. However, the key aspect of this work is its use as an educational tool to engage students. It would be helpful to add some data or at least anecdotal statements to the discussion about the success of the educational goals. For example, do instructors joining the project tend to stay with it in successive years? Is there survey or other data from the students or TAs about whether it’s a valuable aid? Adding some text to the discussion, or results if available, would help other instructors decide if this would be a valuable addition to their curricula.

Is the rationale for developing the new method (or application) clearly explained?

Yes

Is the description of the method technically sound?

Yes

Are the conclusions about the method and its performance adequately supported by the findings presented in the article?

Yes

If any results are presented, are all the source data underlying the results available to ensure full reproducibility?

Yes

Are sufficient details provided to allow replication of the method development and its use by others?

Yes

Reviewer Expertise:

Expertise in manual and automated gene annotation techniques and supporting datasets

I confirm that I have read this submission and believe that I have an appropriate level of expertise to confirm that it is of an acceptable scientific standard, however I have significant reservations, as outlined above.

# Response

**Published:** 2023-06-27

**Authors:** Chinmay Rele

Thank you for your time and detailed descriptions of the ways you think our methods article could be better improved. We have made most of the changes that you have suggested, and have detailed them below.

*While this is a ‘Methods” paper, the authors should perhaps provide some context in the Introduction about what GEP is and what its broader goals are (citing the latest publication on GEP) for the benefit of the naïve reader before diving into the details of the manual annotation protocol.*You suggested to add some context about what the GEP is, and what its broader goals of the GEP are.

**This suggestion is very helpful, and more information about the GEP, its mission statement, and larger scientific publications has been added after the third paragraph of the Introduction.**

- *In figure 1, the Y-axis is labeled as “number of predicted protein-coding genes”, but the legend includes the following statement that implies it’s actually number of isoforms: “Prediction differences can partly be attributed to some algorithms predicting a single isoform in a genomic region (e.g., GeneID), while others predict multiple isoforms per genomic region (e.g., genBlastG, Spaln).” Furthermore, the values for NCBI RefSeq Genes are all in the 20-45k range, when NCBI reports most Drosophila species as having 13-15k protein-coding genes. The authors need to check the data for all annotation programs provided in figure 1 and correct the figure*.

**We have made clear that some predictors predict single transcripts or multiple transcripts per genomic region while providing examples of such predictors in the caption of Figure 1**.

- *In table 1, the authors list “UCSC Assembly” with names that largely aren’t shown in UCSC and don’t correspond to the published assembly names. For example, the D. miranda assembly GCA_000269505.2 is named DroMir_2.2. Where did the name DmirGB2 come from? The authors should clarify the meaning of the first column, and add an additional column with the official name for each assembly since it’s difficult to recognize assembly accessions.*

**Thank you for this suggestion and we apologize for the confusion. We have added the canonical assembly names from NCBI under the “Assembly Name” column, and the new assembly name within the GEP framework has been clarified in the caption of this table**.

- *Many of the assemblies being used by the GEP project have been superseded by newer and higher quality assemblies. Do the authors periodically upgrade to new assemblies, or is this viewed as unnecessary effort for the educational goals of the project?*

**We do intend to use newer assemblies as they become available. Our rate of adoption is delayed by the need to synchronize all curricular materials with updated genome assemblies, but we do this as we are able. We have added this at the end of the third paragraph of Introduction.**

- *To support the statement “As of August 2022, GEP students from 79 institutions have used this annotation protocol to construct 2,101 gene models across 27 Drosophila species.”, it would be useful to provide a supplemental table with the list of gene models curated for this project so far. Additional data on conformance of student models to the final consolidated annotations would also be of interest if readily available.*

**We have updated the date, the number of models, and the number of *Drosophila* species. We have also included a heatmap showing the number of models submitted for each species/gene combination as a supplemental file**. **The outcome of student gene model conformance to the final model is provided in Figure 12, note that many of the models listed in supplement 7 have not yet been reconciled.**

- *In order to aid access to the final gene models, in addition to the TPA submissions the authors could construct a genome browser track available through the remote Track Hub system. This could be updated periodically (e.g. at the end of each semester or year) with additional gene models, and made available through the Track Hub Registry system. While it would be useful to include the availability of this track in the paper if the authors have the resources to generate it, it’s not a requirement for publication.*

**Thank you for this suggestion. We have created a supplemental file (Supplement 8) with all the TrackHub links that have been generated as of March 24, 2023. We will be creating a TrackHub registry. We have added the reference to the supplemental file at the end of ”Reconciliation Process” at the end of the Methods section**.

- *Figure 5: It seems that the isoform names are reversed in the legend. Looking at the ‘User-created Annotations’ track, Akt-PC is the isoform with the longer coding region and not Akt-PE as stated in the legend.*

**Thank you for pointing this error out – it has been corrected to mention that the PC isoform is the isoform with the longer coding region**.

- *Assembly errors: For cases where the student identifiers an error in a target assembly, how is that information conveyed in the TPA records? Is the TPA sequence corrected via a VCF file? What is done with a case like D. pseudoobscura chico from Figure 11?*

**Assembly errors are documented using a VCF file, which is then submitted to NCBI along with the TPA record for the specific model. This has been added to the end of ”Assembly Errors” in the ”Exceptions to the standard annotation workflow” section**. **If the model is in two scaffolds in our assemblies, we opt to complete the reconciliation in a newer (for this instance, we would choose to reconcile the model in the DpseRefSeq1 (GCF_009870125.1)) assembly because our internal tools cannot adequately validate models that are present in two scaffolds.**

- *For the reconciliation process, how would you determine that an isoform is ‘mislabeled’? Is it in comparison to D. melanogaster isoform name? In general, what are the rules to name isoforms in target species?*

**Good question.** **An explanation of how an isoform is defined/named has been added as a new section called Missing/Mislabeled Isoforms at the end of the Most Common Annotation Errors section**.

- *(a)“The annotation effort of the student annotators is of value because the students can outperform computational gene predictors.” - This statement seems a bit misleading unless there is a mention of scale. Computational annotation methods can predict gene and transcript models on a genomic scale, which is not possible by manual annotators (in a reasonable timeframe). It would be reasonable to say that manual annotators can correct errors found in computational predictions and can fill in gaps, as in the example cited in the paper where computational methods could not detect an additional protein isoform due to the use of an upstream non-AUG start codon. If the authors wish to keep the statement, a suggestion is to add something that conveys “at the level of an individual gene or a small set of genes” at the end.*

**Thank you for pointing out this important distinction and lack of elaboration. We have now added the “individual gene level” qualifier to this at the end of the requested sentence**. **We have also pointed out the computational gene prediction algorithms are very valuable at the genome scale.**

- *(b) It would also be helpful to add some statistics on how often students identify errors or additions to automated gene sets.*

**This is an interesting question, and we hope to report on it in a future study, once we have enough data to be statistically robust.**

- *The report is primarily focused on describing aspects of the annotation protocol. However, the key aspect of this work is its use as an educational tool to engage students. It would be helpful to add some data or at least anecdotal statements to the discussion about the success of the educational goals. For example, do instructors joining the project tend to stay with it in successive years? Is there survey or other data from the students or TAs about whether it’s a valuable aid? Adding some text to the discussion, or results if available, would help other instructors decide if this would be a valuable addition to their curricula*.****

**Studies on student learning outcomes showing the benefits to students have been performed by the GEP in separate publications, and a brief description of these findings have been added as the last paragraph of Discussion**.

 Thank you again for all your input to help us make this manuscript more complete and robust.
